# Identification of a Rare Case With Nagashima-Type Palmoplantar Keratoderma and 18q Deletion Syndrome *via* Exome Sequencing and Low-Coverage Whole-Genome Sequencing

**DOI:** 10.3389/fgene.2021.707411

**Published:** 2021-09-20

**Authors:** Qianqian Li, Xiaofan Zhu, Conghui Wang, Jingjing Meng, Duo Chen, Xiangdong Kong

**Affiliations:** Genetics and Prenatal Diagnosis Center, Department of Obstetrics and Gynecology, The First Affiliated Hospital of Zhengzhou University, Zhengzhou, China

**Keywords:** Nagashima-type palmoplantar keratoderma, 18q deletion syndrome, exome sequencing, low-coverage whole-genome sequencing, *SERPINB7*, mosaicism

## Abstract

Nagashima-type palmoplantar keratoderma (NPPK) is characterized by non-progressive, diffuse, and cross-gradient hyperkeratosis caused by mutations in the *SERPINB7* gene on chromosome 18q21.33. Chromosome 18q deletion syndrome (18q- syndrome) is a terminal deletion or microdeletion syndrome characterized by intellectual disability and congenital malformations. This paper describes an 18-year-old man with palmoplantar keratoderma and diffuse white matter abnormalities in the brain. Trio-based exome sequencing (ES) revealed a suspected mosaic compound heterozygous mutation for c.796C>T (p.Arg266^∗^) in exon 8 inherited from the mother and a *de novo* exons 4–6 deletion of *SERPINB7*. Additional copy number variant (CNV) analysis of the ES data indicated a heterozygous gross deletion of 18q22.3-q23. The two *SERPINB7* gene variants were verified by Sanger sequencing and quantitative real-time polymerase chain reaction (qRT-PCR). Finally, low-coverage whole-genome sequencing (WGS) confirmed the 18q22.3-q23 deletion and additionally detected a mosaic 18q21.33-q22.3 deletion, together explaining NPPK and the neurological phenotypes of the proband. The gross deletion of all exons of *SERPINB7* was revealed for the first time. More rarely, c.796C>T (p.Arg266^∗^) was likely to be mosaic, while the exon deletion was mosaic. In conclusion, the combination of multiple molecular genetic testing methods provides comprehensive informative molecular findings and promotes the diagnosis of complex diseases, as in this case.

## Introduction

Nagashima-type palmoplantar keratoderma (NPPK, MIM# 615598) is the most common type of palmoplantar keratoderma in Asian populations (Kubo, 2014), with a prevalence in Japan and China of 1.2/10000 and 3.1/10000, respectively (Kubo et al., 2013). The clinical manifestations of NPPK include mild diffuse palmoplantar hyperkeratosis, diffuse erythema with clear boundaries on the dorsum of the hands, feet, forearm, and elbow, and Achilles tendon and knee. NPPK is caused by homozygous or compound heterozygous mutations in the serpin family B member 7 gene (*SERPINB7*) (Hannula-Jouppi et al., 2020) mapping on chromosome 18q21.33. To date, 14 variants of *SERPINB7* have been incorporated into HGMD^®^ Professional 2021.2 (Supplementary Table 1). More than 90% of patients with NPPK carry the founder nonsense mutation c.796C>T (p.Arg266^∗^). Gross deletions, insertions, complex re-arrangements, and repeats have not yet been reported.

Chromosome 18q deletion syndrome (18q- syndrome, MIM# 601808), a rare autosomal-dominant disorder, is a terminal deficiency or microdeletion syndrome characterized by intellectual disability and congenital malformations (Finley et al., 1969). The clinical symptoms are highly variable, including cognitive impairment from normal intelligence to severe intellectual disability, diffuse white matter abnormalities of the brain (Linnankivi et al., 2003), short stature, delayed myelination (Linnankivi et al., 2006), ear canal abnormalities (Feenstra et al., 2011), genital abnormalities, and foot deformities (Versacci et al., 2005).

This paper presents a rare case of NPPK and diffuse white matter abnormalities in the brain, with dual diagnosis of a suspected mosaic *SERPINB7* gene mutation and an exon deletion along with 18q deletion syndrome *via* trio-based exome sequencing (ES) and low-coverage whole-genome sequencing (WGS).

## Materials and Methods

### Ethics Statement

An affected man with palmoplantar keratoderma and brain white matter abnormality and his family provided written informed consent for genetic studies. The study was approved by the appropriate local institutional review boards on human subject research at the First Affiliated Hospital of Zhengzhou University and conformed to the guidelines set forth by the Declaration of Helsinki.

### DNA Extraction

Genomic DNA from peripheral blood was obtained from 500 μL of whole blood using the Lab-Aid Nucleic Acid (DNA) Isolation Kit (Zeesan, Xiamen, China) in accordance with the manufacturer’s instructions. Genomic DNA from other types of samples, including hair follicle cells, oral swabs, and urine, was extracted using the QIAamp DNA Blood & Tissue Kit (Qiagen, Germany).

### Quantitative Fluorescent Polymerase Chain Reaction

The genetic relationship of the family members was confirmed by quantitative fluorescent polymerase chain reaction (QF-PCR) using the GoldeneyeTM DNA ID System 20A Kit (Peoplespot, Beijing, China).

### Trio-Based Exome Sequencing

Trio-based exome sequencing (trio-ES) was performed using Illumina library construction and capture kits (Illumina, San Diego, CA, United States) in accordance with standard instructions (Document# 1000000048601v03), and 150 bp pair-end sequencing was conducted on NovaSeq 6000 (Illumina).

### Mapping, Variant Calling, and Variant Annotation

The Efficient Genosome Interpretation System (EGIS; Sierra Vast Bio-Medical, Shanghai, China) was used for mapping, variant calling, and variant annotation. The reads were aligned to the hg19/GRCh37 human reference genome sequence.

For single-nucleotide variants (SNVs) and small indels selecting, palmoplantar keratoderma (HP: 0000982) was entered into the EGIS, and 146 OMIM genes (Supplementary Table 2) were obtained based on the human phenotype ontology (Kohler et al., 2019). For CNV selecting, the bpCNV scan tool in the EGIS was used. The background library was constructed by calculating the correlation coefficient (*R* > 0.94) according to the average sequencing depth and exon fragment length of the target sample and reference samples (20 healthy subjects in the same batch). XHMM was used for exon and chromosome CNV calling in the ES data (Fromer et al., 2012). The copy number ratio of exon CNVs was obtained by dividing the exon reads per kilobase per million mapped reads (RPKM) value of the target sample by the average RPKM value of background library samples.

The potential impact of SNVs, small indels, and CNVs was investigated using Ensemble^1^, RefSeq^2^, and other databases, including OMIM^3^, ClinVar^4^, ClinGen^5^, and DECIPHER^6^. The pathogenicity of all variations was evaluated in accordance with the latest guidelines of the American College of Medical Genetics and Genomics (ACMG; Riggs et al., 2020) and the ClinGen Sequence Variant Interpretation (SVI) Workgroup. Finally, polymerase chain reaction (PCR) amplification and Sanger sequencing were performed to confirm the SNV in *SERPINB7* by using the primer pairs listed in Supplementary Table 3.

### Quantitative Real-Time PCR (qRT-PCR)

The DNA samples, including those obtained from the proband, parents, and a healthy control subject (unrelated to this family without any skin diseases), were diluted to 50 ng μL^–1^. qRT-PCR was conducted with KAPA SYBR FAST qPCR Kits (KK4601, Roche, Salt River Cape Town, South Africa) on the ABI QuantStudio5 Real-Time PCR System using the primer pairs exhibited in Supplementary Table 3. Human *GAPDH* was used as a reference gene, and the f fold-change was calculated using the 2^−ΔΔCt^ method.

### Low-Coverage Whole-Genome Sequencing

The experimental methods and data analysis of low-coverage WGS have been previously described (Wang et al., 2018). CNVs (GRCh37.p13) were analyzed and queried against public databases, including DGV^7^, gnomAD^8^, DECIPHER, OMIM, UCSC^9^, and ClinGen. Pathogenicity was assessed according to the latest guidelines outlined by the ACMG (Riggs et al., 2020).

*CopyN*_*bin*_ is the product of *obsRC*_*bin*_ and *refRC*_*bin*_ multiplied by *copyN*_*chrom*_ (*CopyN*_*bin*_, copy number of a bin; *obsRC*_*bin*_, read number of the observed sample in this bin; *refRC*_*bin*_, read number of the reference sample in this bin; *copyN*_*chrom*_, theoretical copy number of the chromosome in this bin). Finally, the mosaic level was assessed based on the copy number value of the chromosomes.

## Results

### Clinical Report

**FIGURE 1:**
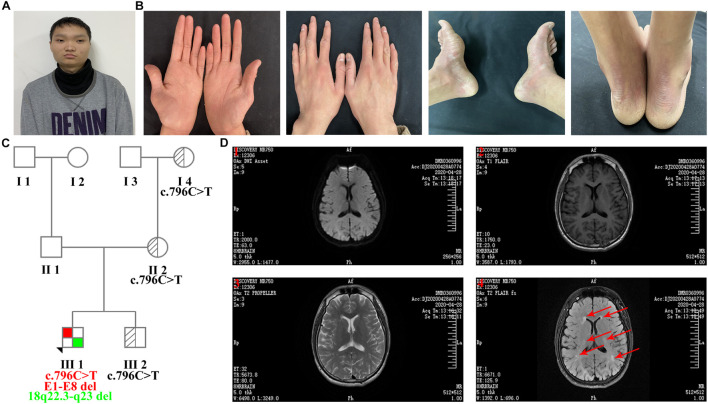
Clinical features of the proband. **(A)** Facial features: wide-set eyes and short philtrum. **(B)** Bilateral erythema and hyperkeratosis of the hands and feet. **(C)** Family tree (E1-E8 del: exon 1-exon 8 deletion). **(D)** MRI images of transverse views. (1) Diffusion-weighted imaging (DWI); (2) T1 weighted image (T1WI); (3) T2 weighted image (T2WI); (4) Water image. MRI images indicated that the proband had multiple abnormal signals in the bilateral frontal and parietal lobes, and red arrows represent the regions of brain white matter abnormality.

The proband was an 18-year-old man with slight facial abnormalities (wide-set eyes and short philtrum) (Figure 1A), mild intellectual disability, and developmental delay. The typical clinical symptoms of mild diffuse palmoplantar hyperkeratosis and diffuse erythema with clear boundaries on the dorsum of the hands and feet and Achilles tendon appeared in the first year after birth (Figure 1B). The palms and soles of the feet had a tendency to peel, and the latter were accompanied by a foot odor. The skin on the sole of the foot was thick and hard, and the skin on the palm of the hand was slightly thinner. These symptoms have been observed since palmoplantar hyperkeratosis was observed. At the age of 10 years, the proband was diagnosed with white matter abnormalities in the brain *via* magnetic resonance imaging (MRI) (data not shown). At the age of 17 years, the proband underwent a brain MRI scan (ID: DMR0360996) in our hospital. MRI images indicated that the proband had multiple abnormal signals in the bilateral frontal and parietal lobes (Figure 1D), which were similar to the previous MRI images, indicating that the abnormal areas of the white matter did not increase. Electrocardiogram (ECG) preformed in the same year revealed that the proband had sinus bradycardia (Supplementary Figure 1). The patient’s parents, sibling, and other family members had no clinical manifestations. The family tree is presented in Figure 1C.

### Trio-ES Results

**FIGURE 2:**
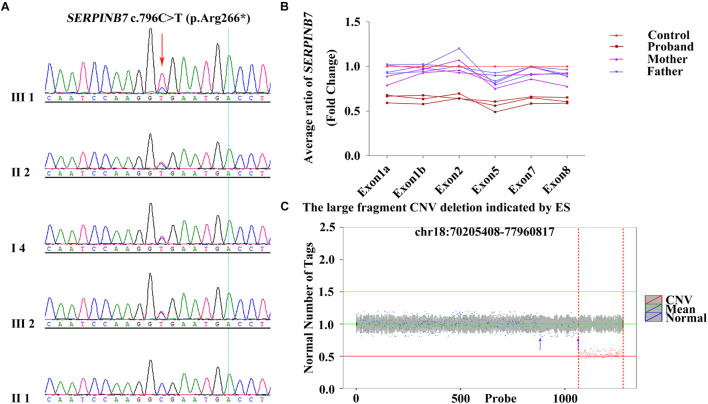
c.796C>T (p.Arg266*) heterozygous variant and exon deletion of *SERPINB7* along with and 18q deletion identified by trio-ES. **(A)** Results of Sanger sequencing confirmed that the heterozygous variant c.796C>T (p.Arg266*) in the proband was inherited from the mother. **(B)** qRT-PCR results demonstrated that exons 1–8 deletion in the proband was a *de novo* deletion. **(C)** Large fragment chromosome CNV deletion indicated by trio-ES in the proband. *X*-axis: the position on the chromosome corresponding to the region of variation currently displayed. The red and blue marks indicate the areas of variant and normal, respectively. *Y*-axis: ratio of target sample RPKM value to the mean value of background library RPKM.

The results of QF-PCR confirmed the genetic relationship among the four family members (Supplementary Tables 4,5 and Supplementary Figures 2,3). The quality control of trio-ES data is revealed in Supplementary Table 6. After filtering, a heterozygous variant of *SERPINB7* (NM_001040147) c.796C>T (p.Arg266^∗^) in exon 8 (reference allele/alternative allele, ref/alt: 5/13) inherited from the mother (ref/alt: 12/28) was identified (Sheet 1 in Supplementary Excel 1 and Supplementary Figures 4–6), and c.796C>T (p.Arg266^∗^) was verified by Sanger sequencing (Figure 2A). Moreover, c.796C>T (p.Arg266^∗^) was further identified in other types of samples from the proband, including hair follicle cells, oral swabs, and urine using Sanger sequencing, and the results were similar to those of the peripheral blood (Supplementary Figure 7). All *de novo* variants (irrespective of the candidate genes) in the proband identified by trio-ES are listed in Supplementary Excel 2.

By analyzing the gene exon-CNV data of trio-ES, the deletion of exons 4–6 of *SERPINB7* was indicated (Sheet 2 in Supplementary Excel 1 and Supplementary Table 7). qRT-PCR was carried out to further confirm whether the exons of *SERPINB7* were deleted. The results of qRT-PCR confirmed that exons 1–8 were all deleted, which was a *de novo* deletion with an average ratio of 0.62 (Figure 2B and Supplementary Table 7). Consequently, c.796C>T (p.Arg266^∗^) and exons 1–8 deletion in *SERPINB7* constituted a compound heterozygous state, leading to the occurrence of NPPK. Moreover, CNV analysis of the ES data indicated an approximately 7.75-Mb heterozygous deletion of 18q22.3-q23 (chr18:70205408-77960817) (Figure 2C and Supplementary Table 8), which was located in the key region of the 18q deletion syndrome, covering 28 coding genes, including 4 OMIM genes: *CYB5A*, *CTDP1*, *TSHZ1*, and *TXNL4A*. More interestingly, the ES-CNV scatter plot (Figure 2C, blue arrow) suggested that the deletion may be larger than 7.75 Mb, which covers the upstream region including *SERPINB7.* Furthermore, the copy number ratios were suggestive of a mosaic state in the upstream region.

### 18q21.33-q22.3 and 18q22.3-q23 Heterozygous Deletions Verified by Low-Coverage Whole-Genome Sequencing

**FIGURE 3:**
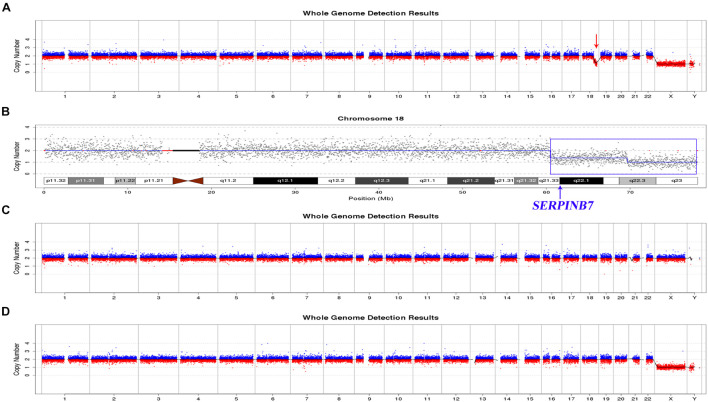
Results of low-coverage WGS of the proband and parents. **(A)**, **(C)**, **(D)** Overview of the low-coverage WGS results of the proband, mother, and father, respectively. The red arrow indicates the region with abnormal copy numbers in the proband. The mother and the father both had normal copy numbers for all the chromosomes. **(B)** Low-coverage WGS results for chromosome 18 of the proband. The blue box shows the 18q21.33-q22.3 mosaic deletion and 18q22.3qter full deletion.

An approximately 9.18-Mb mosaic heterozygous deletion of 18q21.33-q22.3 (chr18: 60480000-69660000) with a mosaic level of ∼40% (Figure 3B and Supplementary Table 7) and an 8.36-Mb heterozygous deletion of 18q22.3-q23 (chr18:69660000-78020000) were identified by low-coverage WGS (Figure 3B and Supplementary Table 8). However, no chromosomal abnormalities were found in the parents (Figure 3), indicating that the gross heterozygous deletions of 18q21.33-q22.3 and 18q22.3-q23 in the proband were both *de novo*.

## Discussion

Nagashima-type palmoplantar keratoderma is an autosomal recessive PPK, and c.796C>T (p.Arg266^∗^) in *SERPINB7* is the most common disease-causing variant in the Asian population. Other types of variants have been rarely reported. In the present family, the trio-ES results suggested that the proband carries the heterozygous variant c.796C>T (p.Arg266^∗^) in exon 8 and deletion of exons 4–6 (confirmed as deletion of all exons by qRT-PCR), which may constitute a compound heterozygous state causing NPPK.

An approximately 9.18-Mb mosaic heterozygous deletion of 18q21.33-q22.3 (chr18:60480000-69660000) with a mosaic level of ∼40% was confirmed by low-coverage WGS, and this region contained 23 OMIM genes, including *SERPINB7* (Supplementary Figure 8). The low-coverage WGS result indicated that the *SERPINB7* whole gene deletion was part of the larger 9.18-Mb mosaic deletion. Moreover, the large CNV (18q22.3-q23 deletion at chr18:70205408-77960817) identified by trio-ES was confirmed by low-coverage WGS (deletion at 18:69660000-78020000), although the boundaries of the CNV regions were slightly different.

The ES ref/alt ratio and the Sanger sequencing results are not conclusive about the mosaic state of c.796C>T (p.Arg266^∗^), since the decreased representation of mutant alleles could be due to technical reasons. However, the c.796C>T (p.Arg266^∗^) homozygous variant should be detected if the whole exons of *SERPINB7* are deleted. The fact that the *SERPINB7* deletion is mosaic (for which on the contrary, the data are convincing) could explain the fact that some wild-type (wt) alleles were detected. This indicates that c.796C>T (p.Arg266^∗^) is probably present in a mosaic form. An important limitation of this study is that no direct evidence could prove the mosaic state of c.796C>T (p.Arg266^∗^). Considering that c.796C>T (p.Arg266^∗^) was inherited from the mother, we speculated that three cell populations of wt + wt, wt + deletion, and wt + c.796C>T (p.Arg266^∗^) were present in the proband. However, this would not represent a compound heterozygosity and, therefore, would not explain the phenotype of NPPK. In addition, for the proband, the heterozygosity states of c.796C>T (p.Arg266^∗^) in hair follicle cells, oral swabs, and urine were similar to those in the peripheral blood (Supplementary Figure 7). Therefore, c.796C>T (p.Arg266^∗^) may be mosaic, and together with the mosaic exon deletion, result in NPPK in the proband. However, if the mechanism by which a germline-inherited variant of c.796C>T (p.Arg266^∗^) can become mosaic does exist, it would be much less likely than a situation in which the inherited c.796C>T (p.Arg266^∗^) is present in all cells (heterozygous) and only the deletion at chr18:60480000-69660000 is in a mosaic state. Although we cannot demonstrate this, it is a possible scenario.

In this study, the gross deletion of eight exons in *SERPINB7* was revealed for the first time. The observation that this exon deletion in *SERPINB7* was mosaic is highly rare. Although c.796C>T (p.Arg266^∗^) may also be mosaic, there was no direct evidence to prove its mosaic form. Moreover, the exact mosaic level of the 9.18-Mb heterozygous deletion will need to be investigated further in future studies, such as fluorescence *in situ* hybridization. Although the autosomal recessive inheritance pattern of one of the SNV in one allele and the other of CNV in other allele has been reported in other types of diseases (Miksch et al., 2005; Miousse et al., 2011), this has yet to be reported for NPPK.

Furthermore, the proband was found to have *de novo* deletions of the 18q21.33-q22.3 region (chr18:60480000-69660000) in a mosaic state and the 18q22.3qter region (chr18:69660000-78020000) in full form by low-coverage WGS. The DECIPHER database includes similar cases with overlapping deletions. Case ID (272505) (chr18:60679560-68497696) presented global developmental delay, protruding ears, and short stature, whereas no phenotype was recorded for Case ID (254160, *de novo*) (chr18:63567516-70799806). Case ID (248930, *de novo*) (chr18:69191420-78014582) showed small nails, abnormal toes, and frontal bossing. Case ID (267134, *de novo*) (chr18:69199778-77982186) showed delayed puberty, proportionate short stature, ptosis, and stenosis of the external auditory canal. However, the DGV database does not include cases with similar CNV fragment deletions (chr18:60480000-69660000 and chr18:69660000-78020000). Therefore, it is possible that the two deletions might together contribute to the neurological phenotype of the proband. Interestingly, the 18q22.3qter deletion was indicated by the ES data, but the 18q21.33-q22.3 mosaic deletion was missed. Although the scatter plot of this region (Figure 2C) was slightly skewed from the mean copy number, this finding suggests that low-coverage WGS is more sensitive in the detection of CNV mosaicism (> 30%) than ES. In combination with the *SERPINB* gene variants, the results of low-coverage WGS provide accurate information for the genetic counseling and management of the patient, including the possibility of prenatal and pre-implantation diagnosis in the future.

Exome sequencing is a state-of-the-art method that enables the direct assessment of variants in the protein-coding region, which has been successfully used as a diagnostic approach to investigate the underlying genetic etiology of complex phenotypes (Tammimies et al., 2015). CNVs range in size from changes of a few hundred base pairs to enlargements or deletions of millions of base pairs of DNA (Conrad et al., 2010). Large CNVs are detected in severe pediatric cases, including neurological and congenital birth defects and neuropsychiatric disorders. ES may indicate the existence of large fragment deletions, and low-coverage WGS can verify the results (Bradshaw et al., 2018) and even uncover additional findings, as shown in the case presented in this study. The combined use of ES and low-coverage WGS may provide comprehensive genetic information for the molecular diagnosis of complex single- or multi-system diseases.

In conclusion, we report a rare case of NPPK and brain white matter abnormalities caused by *SERPINB7* mutations and 18q deletion syndrome. This study demonstrated that the combination of multiple genetic testing methods allowed for the accurate diagnosis of complex diseases.

## Data Availability Statement

Publicly available datasets were analyzed in this study. This data can be found here: the data supporting the findings of this research are available within the article/Supplementary Material.

## Ethics Statement

The studies involving human participants were reviewed and approved by the appropriate local institutional review boards on human subject research at the First Affiliated Hospital of Zhengzhou University and also conformed to the guidelines set forth by the Declaration of Helsinki. The proband and his family provided written informed consent to participate in this research. The project was conducted in accordance with the International Ethical Guidelines for Biomedical Research Involving Human Subjects (CIOMS). Written informed consent to participate in this study was provided by the participants’ legal guardian/next of kin. Written informed consent was obtained from the individual(s) for the publication of any potentially identifiable images or data included in this manuscript.

## Author Contributions

QL, XZ, and XK designed the research and wrote the manuscript. QL, XZ, and JM carried out the experiments and data analysis. CW and DC revised the manuscript. All authors contributed to the article and approved the submitted version.

## Conflict of Interest

The authors declare that the research was conducted in the absence of any commercial or financial relationships that could be construed as a potential conflict of interest.

## Publisher’s Note

All claims expressed in this article are solely those of the authors and do not necessarily represent those of their affiliated organizations, or those of the publisher, the editors and the reviewers. Any product that may be evaluated in this article, or claim that may be made by its manufacturer, is not guaranteed or endorsed by the publisher.

## Footnotes

1
http://grch37.ensembl.org/index.html

2
https://www.ncbi.nlm.nih.gov/refseq/

3
https://omim.org/about

4
https://www.ncbi.nlm.nih.gov/clinvar/

5
https://www.clinicalgenome.org/

6
https://www.Deciphergenomics.org/

7
http://dgv.tcag.ca/dgv/app/home

8
https://www.nature.com/collections/afbgiddede/gnomad-website

9
http://genome.ucsc.edu/

## Supplementary Material

Supplementary Excel 1 Sheet 1: Detailed information of c.796C>T (p.Arg266^∗^) in *SERPINB7* identified by trio-ES. Sheet 2: Exon-CNV results of *SERPINB7* for the proband, mother, and father identified by trio-ES. Left: For the proband, exons 4–6 may be deleted. Middle: For mother, exon 5 may be duplicated. Right: For father, the copy number ratio may be normal.

Supplementary Excel 2 All *de novo* variants (irrespective of candidate genes) in the proband identified by trio-ES.
