# Supplementary material for: Identification of a Rare Case With Nagashima-Type Palmoplantar Keratoderma and 18q Deletion Syndrome via Exome Sequencing and Low-Coverage Whole-Genome Sequencing
Source: Front Genet. 2021 Sep 20;12:707411. doi: 10.3389/fgene.2021.707411 (PMC8488357; doi:10.3389/fgene.2021.707411)

**Supplementary Table 1. Variants of *SERPINB7*.**

| **Mutation Type** | **Resources** | **Total Number** | **Codon Change** | **Amino Acid Change** | **Nucleotide** | **Protein** | **Variant Class** | **Reported phenotype** |
| --- | --- | --- | --- | --- | --- | --- | --- | --- |
| Missense/Nonsense | HGMD | 6 | CGA-TGA | Arg128* | c.382C>T | p.R128* | DM | NPPK |
|  |  |  | GGC-GTC | Gly152Val | c.455G>T | p.G152V | DM | NPPK |
|  |  |  | CGA-TGA | Arg266* | c.796C>T | p.R266* | DM | NPPK |
|  |  |  | CCT-CTT | Pro277Leu | c.830C>T | p.P277L | DM | NPPK |
|  |  |  | ATT-ACT | Ile310Thr | c.929T>C | p.I310T | DM? | Autism |
|  |  |  | TGC-TAC | Cys379Tyr | c.1136G>A | p.C379Y | DM | NPPK |
| Splicing |  | 2 | - | - | c.336+2T>G |  | DM | NPPK |
|  |  |  | - | - | c.455-1G>A |  |  | NPPK |
| Regulatory |  | 0 | - | - | - | - | - | - |
| Small deletions |  | 4 |  |  | c.122_127del | p.L41-V42del | DM | NPPK |
|  |  |  |  |  | c.271del | p.H91Tfs*9 | DM | NPPK |
|  |  |  |  |  | c.636del | p.K213Sfs*12 | DM | NPPK |
|  |  |  |  |  | c.650_653del | p.S271Lfs*7 | DM | NPPK |
| Small insertions |  | 1 |  |  | c.522dupT | p.V175Cfs*46 | DM | NPPK |
| Small indels |  | 1 |  |  | c.218_219delAGins12 | p.E73Lfs*17 | DM | NPPK |
| Gross insertions |  | 0 | - | - | - | - | - | - |
| Complex |  | 0 | - | - | - | - | - | - |
| Repeats |  | 0 | - | - | - | - | - | - |
| **Gross deletions** | **This report** | **1** | **-** | **-** | **Exon1-8** | **-** | **DM** | **NPPK** |
| Total |  | 15 |  |  |  |  |  |  |

**Supplementary Table 2. Candidate genes related to palmoplantar keratosis.**

| *DOLK* | *TGM1* | *AAAS* | *CTC1* | *AKT1* | *TNNC1* | *PKP1* | *LEMD3* | *TAT* | *CERS3* |
| --- | --- | --- | --- | --- | --- | --- | --- | --- | --- |
| *USB1* | *WNT10A* | *DSC2* | *JUP* | *PERP* | *NEXN* | *PARN* | *TRPV3* | *AAGAB* | *CD28* |
| *ALOXE3* | *CTSC* | *ENPP1* | *SMARCAD1* | *FGFR2* | *SERPINB7* | *KRAS* | *RSPO1* | *MBTPS2* | *KLLN* |
| *SNAP29* | *PTEN* | *CSRP3* | *DMD* | *ATP2A2* | *DES* | *NOP10* | *NHP2* | *LAMB3* | *LORICRIN* |
| *TNNT2* | *NEBL* | *PEPD* | *CAP2* | *PNPLA1* | *MYPN* | *TTN* | *TRPM4* | *TXNRD2* | *CTLA4* |
| *DSP* | *PLEC* | *LAMC2* | *MYH6* | *GATAD1* | *PRDM16* | *GJA1* | *ABCA12* | *TNFRSF1B* | *KRT17* |
| *KRT16* | *RHBDF2* | *KRT14* | *MYBPC3* | *RBM20* | *PIK3CA* | *SCN5A* | *BAG3* | *SGCD* | *ACTC1* |
| *KRT2* | *KRT1* | *KRT5* | *FKTN* | *KRT9* | *RAF1* | *HPGD* | *TP63* | *NLRP1* | *MYH7* |
| *SEC23B* | *USF3* | *PSEN2* | *PSEN1* | *KRT83* | *TPM1* | *FHL2* | *CAST* | *KRT6B* | *KRT6C* |
| *KRT6A* | *SRD5A3* | *TRAPPC11* | *DSG1* | *ALOX12B* | *TAFAZZIN* | *LAMA3* | *LAMA4* | *CSTA* | *GMPPA* |
| *KDSR* | *DSG2* | *FERMT1* | *COL17A1* | *TCAP* | *CYP4F22* | *TAF1A* | *PLN* | *TRNS1* | *TMPO* |
| *CRYAB* | *MAP2K2* | *MAP2K1* | *HAND2* | *POMP* | *ANKRD1* | *GJB2* | *TERC* | *GJB4* | *GJB6* |
| *TNNI3* | *TERT* | *BRAF* | *ITGB4* | *KRT10* | *VCL* | *SLCO2A1* | *CARD14* | *MCOLN1* | *WRAP53* |
| *RTEL1* | *NPM1* | *SASH1* | *ACTN2* | *NIPAL4* | *LDB3* | *TINF2* | *COL14A1* | *KANK2* | *ABCC9* |
| *DKC1* | *SDHA* | *SDHC* | *SDHB* | *SDHD* | *PPCS* |  |  |  |  |

**Supplementary Table 3. Primer sequences of Sanger sequencing and qRT-PCR for *SERPINB7.***

| **Primer name** | **Primer sequence (Forward)** | **Primer sequence (Reverse)** |
| --- | --- | --- |
| *SERPINB7*-Sanger Sequencing | 5′-CTGCCCAAGACACATTCATG-3′ | 5′-TGGGTGGTCAGCTCTAAACAG-3′ |
| *SERPINB7*-Exon1a-qRT-PCR | 5′-GGAAGGATGAGCAACACGAC-3′ | 5′-CTTCATCCCAGACTGCACAA-3′ |
| *SERPINB7*-Exon1b-qRT-PCR | 5′-TGGGAATTTTCCCTGAACCT-3′ | 5′-GGGCTGTCCATCCATCTTAGG-3′ |
| *SERPINB7-*Exon2-qRT-PCR | 5′-CCGGGAACTAATTTCATTTTCTC-3′ | 5′-TCCCAGTGATTAACTGCAAAAG-3′ |
| *SERPINB7-* Exon5-qRT-PCR | 5′-GGTGATGATTTGTAAATACGAGAA-3′ | 5′-TGACAAATCTTGTAGAAAAATAGGG-3′ |
| *SERPINB7-* Exon7-qRT-PCR | 5′-AAAGTAAAATGAGGTGGGATCA-3′ | 5′-CCCAATAGTGAAAATGACAGTCG-3′ |
| *SERPINB7-* Exon8-qRT-PCR | 5′-TCATGGGTGAGCAGAGAGTG-3′ | 5′-TGAATGTGTCTTGGGCAGTC-3′ |

**Supplementary Table 4. The STR locus of Ⅲ 1.**

| **STR locus** | **Ⅱ 2** | | **Ⅲ 1** | | **Ⅱ 1** | | **Father gene 1** | **Father gene 2** | **Calculation formula** | **PI** |
| --- | --- | --- | --- | --- | --- | --- | --- | --- | --- | --- |
| D19S433 | 15.2 | 16.2 | 13 | 16.2 | 13 | 13 | 0.2313 |  | 1/(2*p) | 23.4742 |
| D5S818 | 10 | 12 | 7 | 10 | 7 | 10 | 0.0213 |  | 1/(2*p) | 4.0128 |
| D21S11 | 29 | 29 | 29 | 32.2 | 31.2 | 32.2 | 0.1246 | 0.1312 | 1/[2*(p+q)] | 1.5547 |
| D18S51 | 13 | 16 | 13 | 16 | 13 | 18 | 0.1904 |  | 1/(2*p) | 3.3113 |
| D6S1043 | 14 | 19 | 19 | 19 | 13 | 19 | 0.1510 | 0.2062 | 1/[2*(p+q)] | 0.9066 |
| D3S1358 | 15 | 17 | 15 | 17 | 16 | 17 | 0.3453 |  | 1/(2*p) | 3.4916 |
| D13S317 | 9 | 10 | 10 | 10 | 10 | 11 | 0.1432 |  | 1/p | 2.8810 |
| D7S820 | 12 | 13 | 11 | 13 | 11 | 11 | 0.3471 |  | 1/(2*p) | 3.9124 |
| D16S539 | 11 | 13 | 10 | 13 | 10 | 12 | 0.1278 |  | 1/p | 4.1102 |
| CSF1PO | 11 | 13 | 10 | 11 | 10 | 10 | 0.2433 |  | 1/(2*p) | 3.7651 |
| Penta D | 9 | 9 | 9 | 12 | 12 | 13 | 0.1328 |  |  |  |
| AMEL | 0 | 0 | 0 | 1 | 0 | 1 |  | 0.1947 | 1/[2*(p+q)] | 1.1606 |
| vWA | 17 | 18 | 17 | 18 | 14 | 18 | 0.2361 | 0.1852 | 1/[2*(p+q)] | 1.2276 |
| D8S1179 | 13 | 14 | 13 | 14 | 14 | 15 | 0.2221 |  | 1/p | 3.3478 |
| TPOX | 8 | 9 | 9 | 11 | 11 | 11 | 0.2987 |  | 1/(2*p) | 2.5667 |
| Penta E | 12 | 19 | 11 | 12 | 11 | 13 | 0.1948 | 0.5215 | 1/(p+q) | 1.2692 |
| TH01 | 7 | 9 | 7 | 9 | 9 | 9 | 0.2664 |  | 1/(2*p) | 2.6302 |
| D12S391 | 19 | 22 | 18 | 19 | 18 | 24 | 0.1901 | 0.2031 | 1/[2*(p+q)] | 2.0000 |
| D2S1338 | 17 | 23 | 17 | 23 | 18 | 23 | 0.0469 |  | 1/(2*p) | 4.6642 |
| FGA | 19 | 23 | 21 | 23 | 21 | 22 | 0.1072 |  | 1/(2*p) | 23.4742 |
|  |  |  |  |  |  |  |  | PI (Accumulation) 441364417.1494 | | |
|  |  |  |  |  |  |  |  | RCP 0.9999999977 | | |

**Supplementary Table 5. The STR locus of Ⅲ 2.**

| STR locus | Ⅱ 2 | | Ⅲ 2 | | Ⅱ 1 | | Father gene 1 | Father gene 2 | Calculation formula | PI |
| --- | --- | --- | --- | --- | --- | --- | --- | --- | --- | --- |
| D19S433 | 15.2 | 16.2 | 13 | 15.2 | 13 | 13 | 0.2313 |  | 1/p | 4.3234 |
| D5S818 | 10 | 12 | 7 | 12 | 7 | 10 | 0.0213 |  | 1/(2*p) | 23.4742 |
| D21S11 | 29 | 29 | 29 | 32.2 | 31.2 | 32.2 | 0.1246 |  | 1/(2*p) | 4.0128 |
| D18S51 | 13 | 16 | 16 | 18 | 13 | 18 | 0.0455 |  | 1/(2*p) | 10.9890 |
| D6S1043 | 14 | 19 | 13 | 19 | 13 | 19 | 0.1328 |  | 1/(2*p) | 3.7651 |
| D3S1358 | 15 | 17 | 16 | 17 | 16 | 17 | 0.3277 |  | 1/(2*p) | 1.5258 |
| D13S317 | 9 | 10 | 10 | 10 | 10 | 11 | 0.1432 |  | 1/(2*p) | 3.4916 |
| D7S820 | 12 | 13 | 11 | 13 | 11 | 11 | 0.3471 |  | 1/p | 2.8810 |
| D16S539 | 11 | 13 | 10 | 13 | 10 | 12 | 0.1278 |  | 1/(2*p) | 3.9124 |
| CSF1PO | 11 | 13 | 10 | 11 | 10 | 10 | 0.2433 |  | 1/p | 4.1102 |
| Penta D | 9 | 9 | 9 | 13 | 12 | 13 | 0.1012 |  | 1/(2*p) | 4.9407 |
| AMEL | 0 | 0 | 0 | 1 | 0 | 1 |  |  |  |  |
| vWA | 17 | 18 | 17 | 18 | 14 | 18 | 0.2361 | 0.1947 | 1/[2*(p+q)] | 1.1606 |
| D8S1179 | 13 | 14 | 13 | 15 | 14 | 15 | 0.1712 |  | 1/(2*p) | 2.9206 |
| TPOX | 8 | 9 | 8 | 11 | 11 | 11 | 0.2987 |  | 1/p | 3.3478 |
| Penta E | 12 | 19 | 12 | 13 | 11 | 13 | 0.0674 |  | 1/(2*p) | 7.4184 |
| TH01 | 7 | 9 | 9 | 9 | 9 | 9 | 0.5215 |  | 1/p | 1.9175 |
| D12S391 | 19 | 22 | 18 | 22 | 18 | 24 | 0.1901 |  | 1/(2*p) | 2.6302 |
| D2S1338 | 17 | 23 | 18 | 23 | 18 | 23 | 0.1438 |  | 1/(2*p) | 3.4771 |
| FGA | 19 | 23 | 21 | 23 | 21 | 22 | 0.1072 |  | 1/(2*p) | 4.6642 |
|  |  |  |  |  |  |  |  | PI (Accumulation) 141483269436.5509 | | |
|  |  |  |  |  |  |  |  | RCP 1.0000000000 | | |

**Supplementary Table 6. The quality control of trio-ES data.**

| **20×Coverage** | | | **Data size (Mb)** | | | **Capture efficiency** | | | **Duplication rate (%)** | | | **Average sequencing depth** | | |
| --- | --- | --- | --- | --- | --- | --- | --- | --- | --- | --- | --- | --- | --- | --- |
| Ⅱ 1 | Ⅰ 1 | Ⅰ 2 | Ⅱ 1 | Ⅱ 2 | Ⅲ 1 | Ⅱ 1 | Ⅱ 2 | Ⅲ 1 | Ⅱ 1 | Ⅱ 2 | Ⅲ 1 | Ⅱ 1 | Ⅱ 2 | Ⅲ 1 |
| 98.99% | 98.48% | 98.93% | 12985 | 11070 | 12024 | 70.08% | 69.23% | 71.48% | 0.172 | 0.157 | 0.163 | 140.98 | 119.44 | 133.12 |

**Supplementary Table 7. Details of the mosaic deletion.**

| Region | Genomic coordinate | Size | Copy number ratio/Relative ratio/Copy Number | CNV type | Detection method |
| --- | --- | --- | --- | --- | --- |
| *SERPINB7* (Exon 4/5/6) | - | - | 0.57/0.62/0.58 | Heterozygous deletion | Trio-ES  (Exon-CNV) |
| *SERPINB7*  (Exon 1/2/5/7/8) |  |  | 0.65/0.66/0.55/0.63/0.61 |  | qRT-PCR |
| (18q21.33-q22.3) | chr18: 60480000-69660000 | 9.18-Mb | 1.4 |  | Low-coverage WGS |

**Supplementary Table 8. Details of the heterozygous deletion.**

| Region | Genomic coordinate | Size | Copy number | CNV type | Detection method |
| --- | --- | --- | --- | --- | --- |
| 18q22.3-q23 | chr18:70205408-77960817 | 7.75-Mb | 1 | Heterozygous deletion | Trio-ES  (Exon-CNV) |
| 18q22.3-q23 | chr18:69660000-78020000 | 8.36-Mb |  |  | Low-coverage WGS |

**Supplementary Figure 1. Electrocardiogram (ECG) of the proband.**


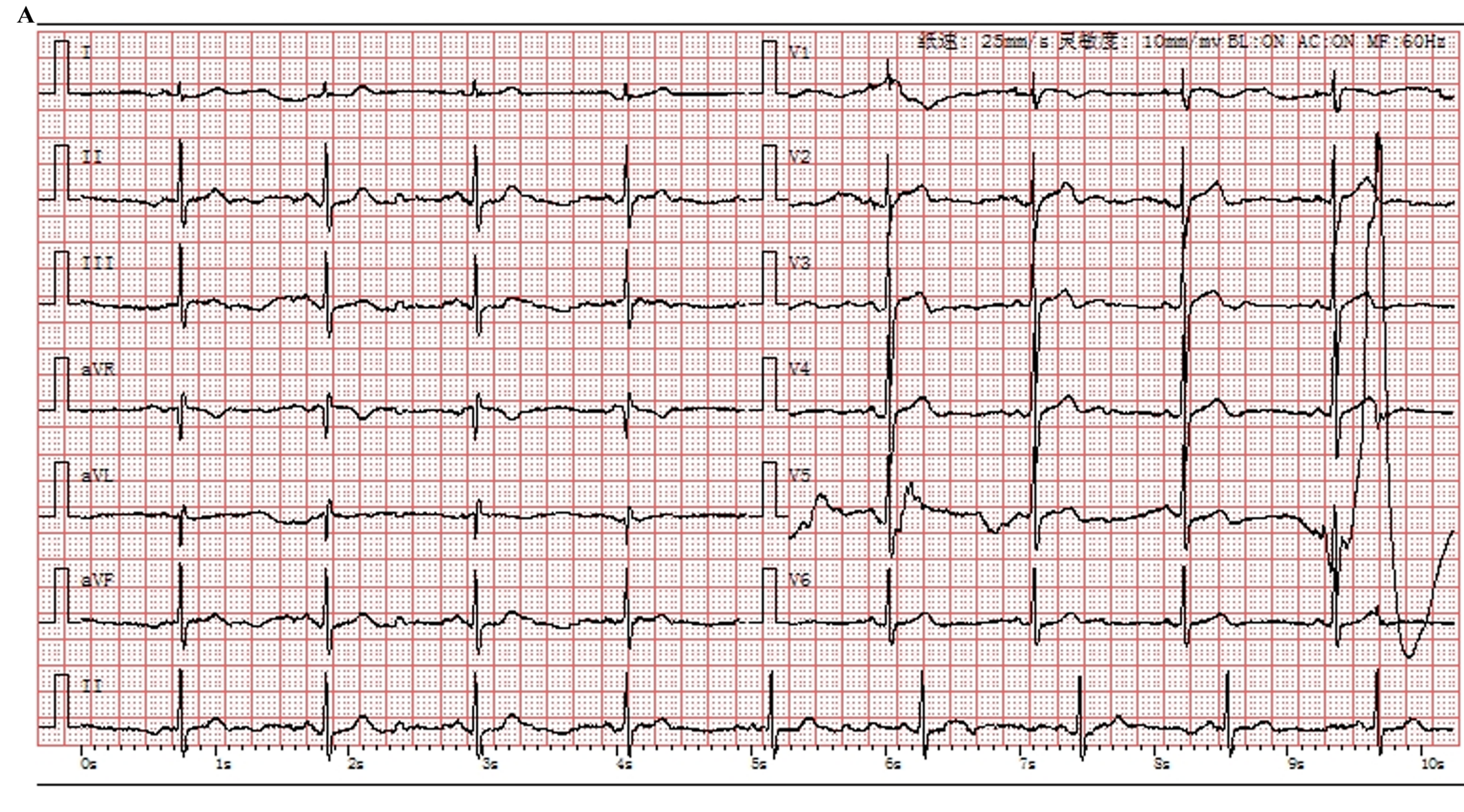


**Supplementary Figure 2. QF-PCR results of Ⅱ1 (A) and Ⅱ2 (B).**

**
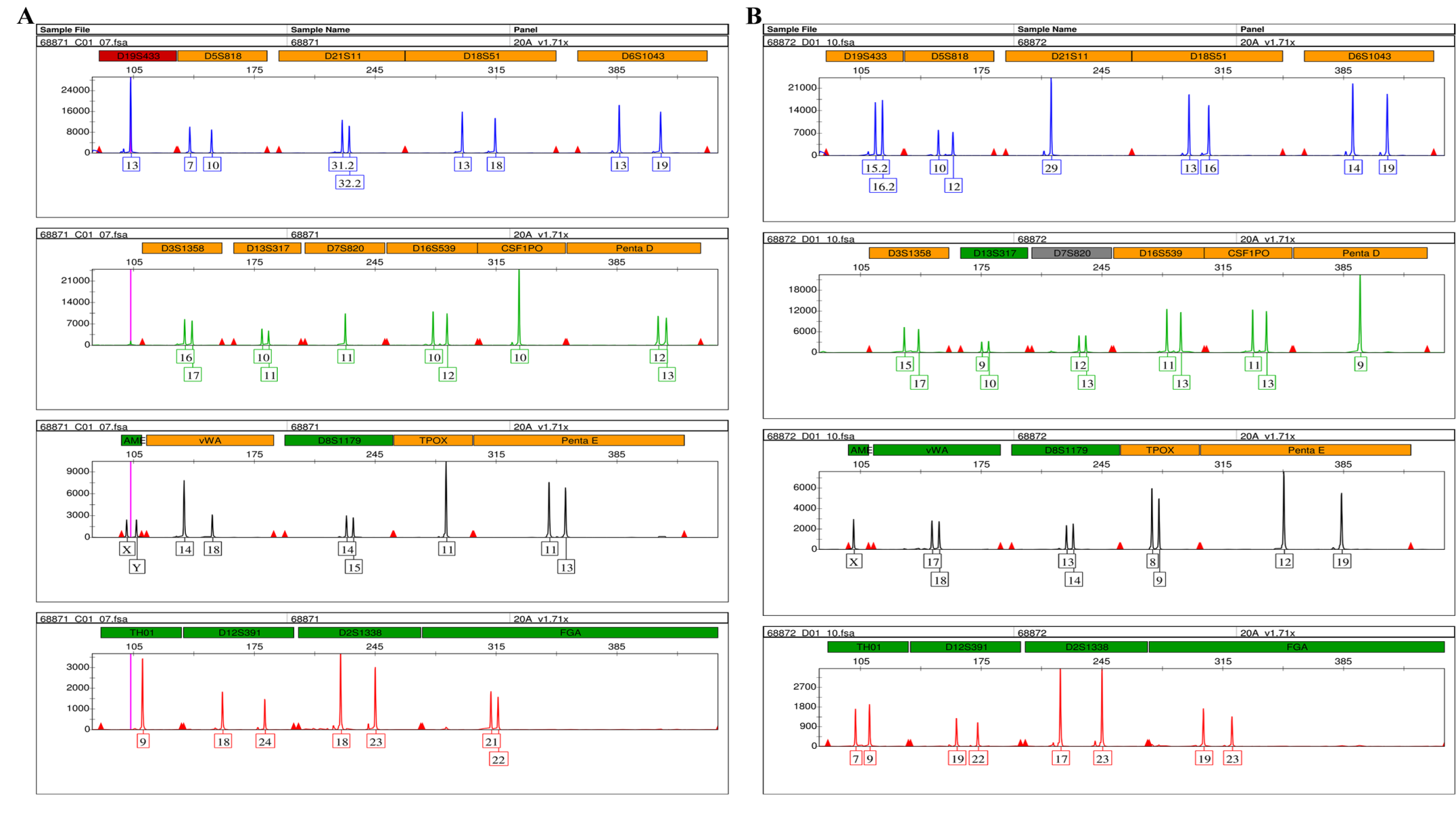
**

**Supplementary Figure 3. QF-PCR results of Ⅲ1 (A) and the Ⅲ2 (B).**

**
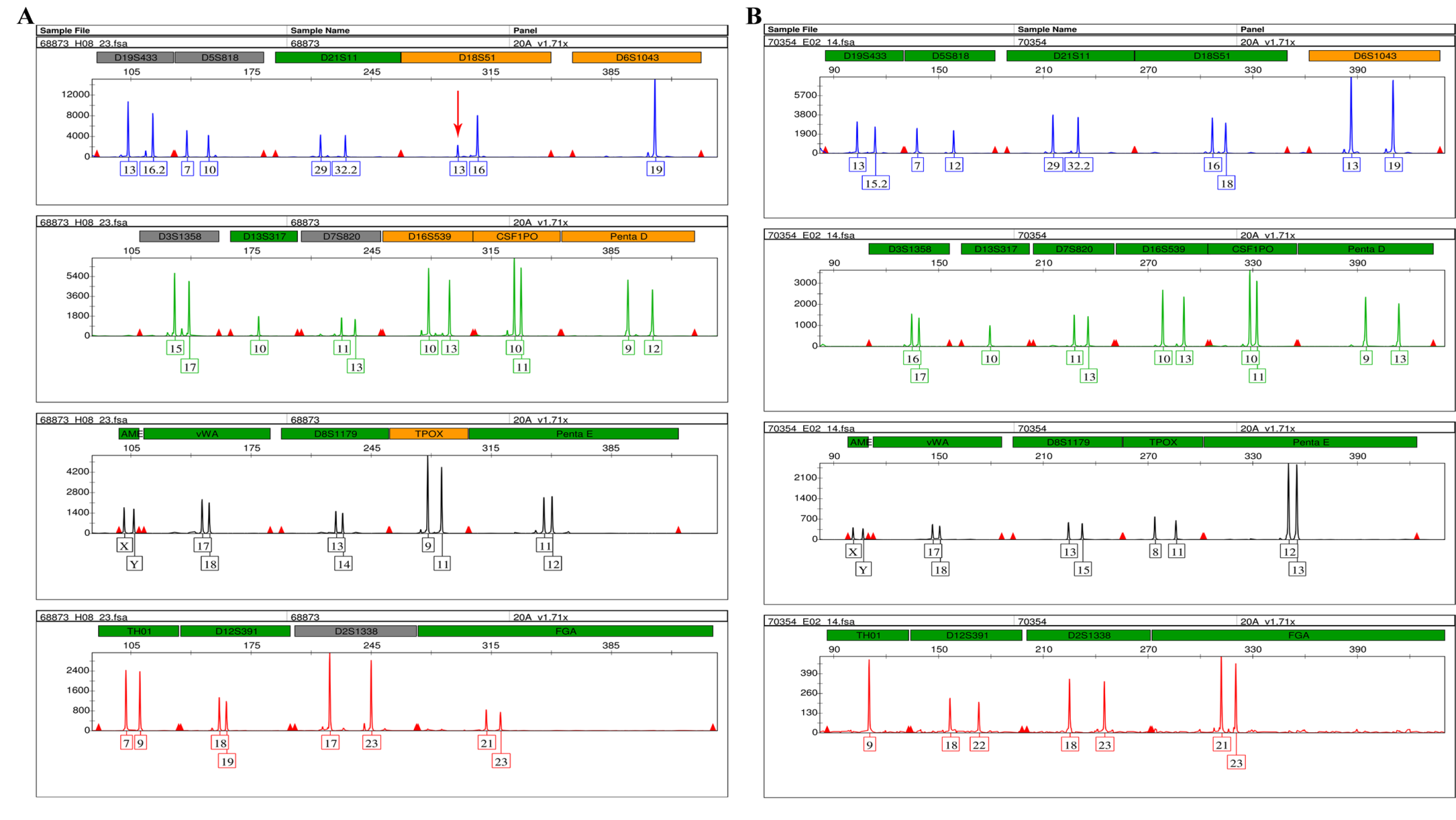
**

**Supplementary Figure 4. Screenshot of the exome alignment of c.796C>T (p.Arg266*) in the proband (the yellow line).**


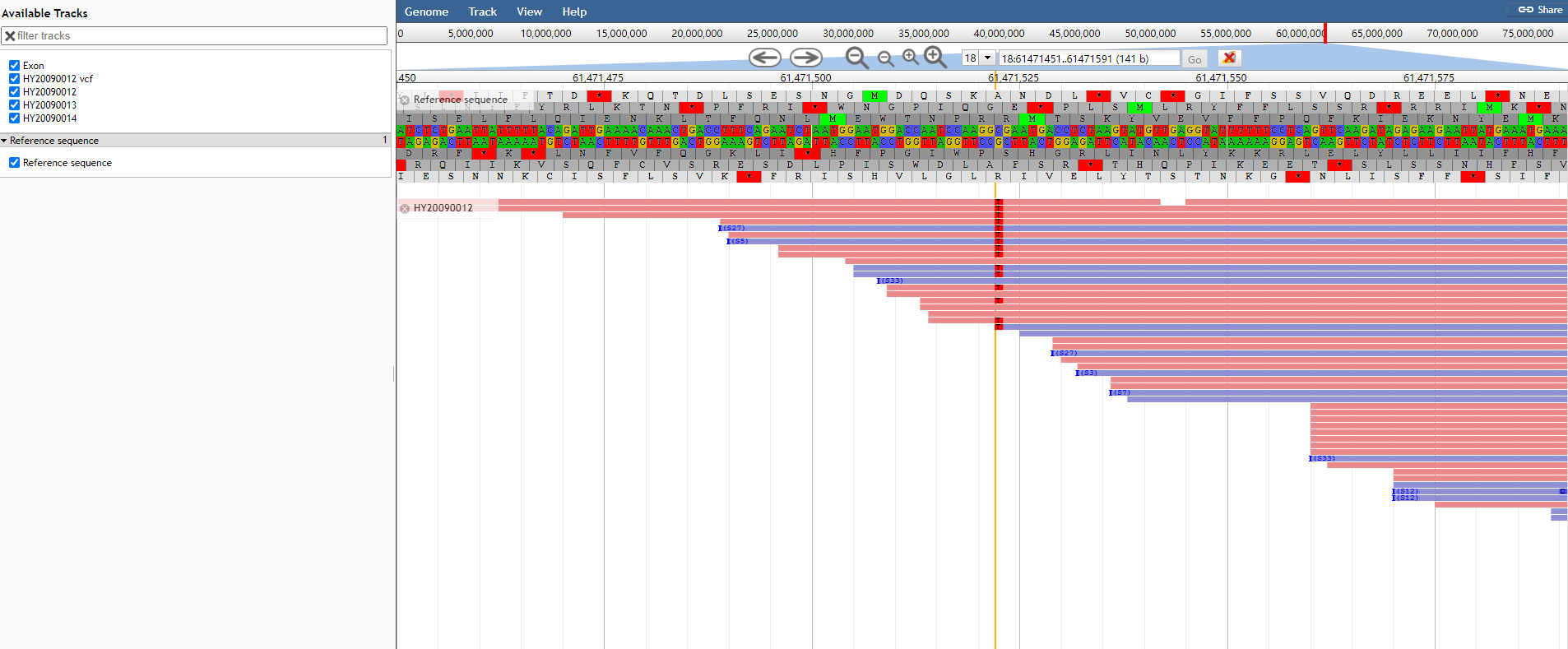


**Supplementary Figure 5. Screenshot of the exome alignment of c.796C>T (p.Arg266*) in the mother (the yellow line).**


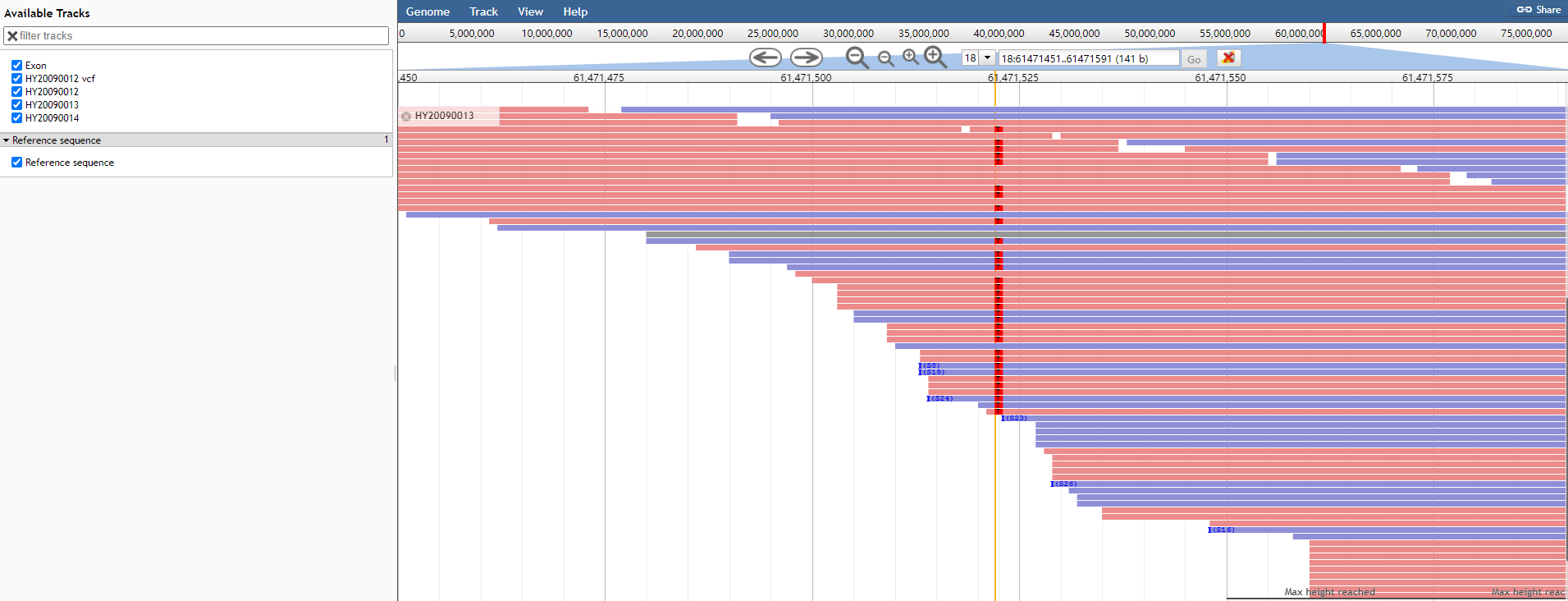


**Supplementary Figure 6. Screenshot of the exome alignment of c.796C>T (p.Arg266*) in the father (the yellow line).**


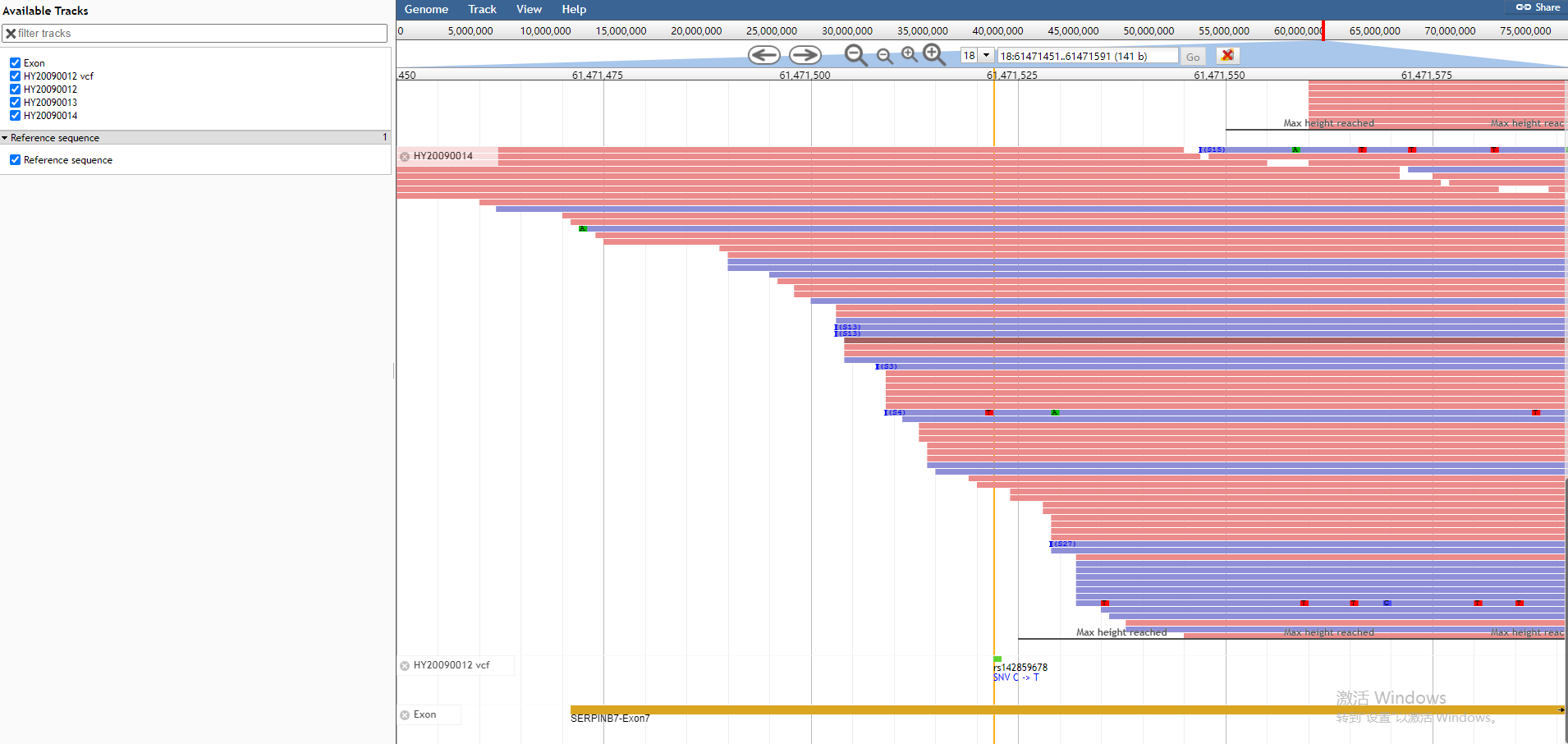


**Supplementary Figure 7. Sanger sequencing results of c.796C>T (p.Arg266*) in hair follicle cells, oral swabs, and urine (red arrow) of the proband.**


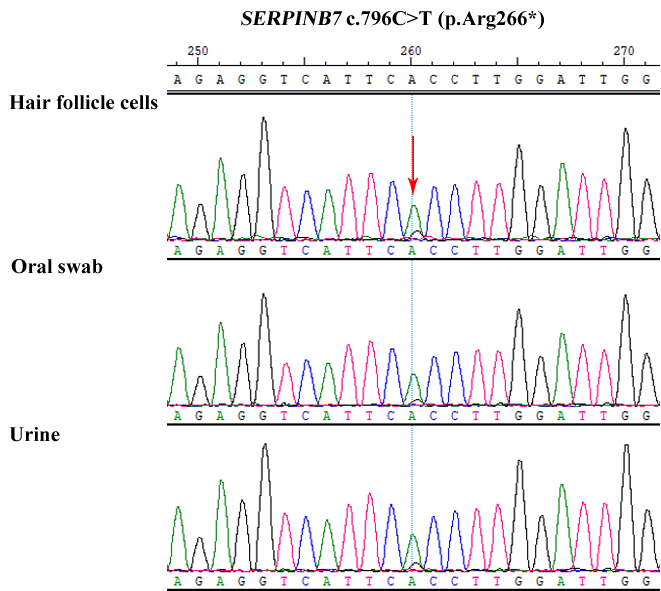


**Supplementary Figure 8. The genes included in chr18:60480000-69660000.**


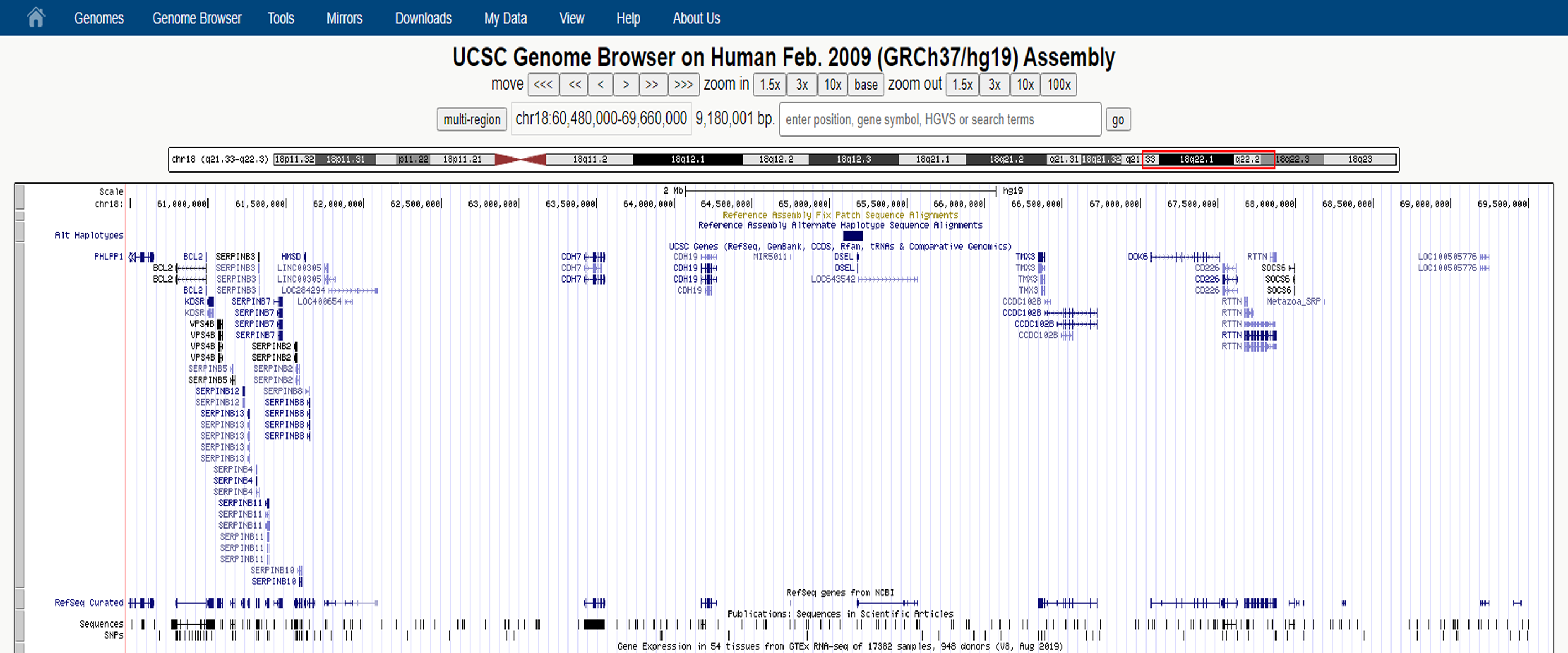

Supplement: Supplementary Excel 1 — Sheet 1: Detailed information of c.796C>T (p.Arg266∗) in SERPINB7 identified by trio-ES. Sheet 2: Exon-CNV results of SERPINB7 for the proband, mother, and father identified by trio-ES. Left: For the proband, exons 4–6 may be deleted. Middle: For mother, exon 5 may be duplicated. Right: For father, the copy number ratio may be normal. [file Data_Sheet_1.zip › All the Supplementary Materials-2021-09-08/Supplementary Tables and Figures.docx]
